# Vitamin D3 Suppresses Class II Invariant Chain Peptide Expression on Activated B-Lymphocytes: A Plausible Mechanism for Downregulation of Acute Inflammatory Conditions

**DOI:** 10.1155/2016/4280876

**Published:** 2016-05-30

**Authors:** Omar K. Danner, Leslie R. Matthews, Sharon Francis, Veena N. Rao, Cassie P. Harvey, Richard P. Tobin, Ken L. Wilson, Ernest Alema-Mensah, M. Karen Newell Rogers, Ed W. Childs

**Affiliations:** ^1^Morehouse School of Medicine, Atlanta, GA, USA; ^2^Texas A&M School of Medicine, Temple, TX, USA; ^3^Michigan State University College of Human Medicine, Flint, MI, USA

## Abstract

Class II invariant chain peptide (CLIP) expression has been demonstrated to play a pivotal role in the regulation of B cell function after nonspecific polyclonal expansion. Several studies have shown vitamin D3 helps regulate the immune response. We hypothesized that activated vitamin D3 suppresses CLIP expression on activated B-cells after nonspecific activation or priming of C57BL/6 mice with CpG. This study showed activated vitamin D3 actively reduced CLIP expression and decreased the number of CLIP^+^ B-lymphocytes in a dose and formulation dependent fashion. Flow cytometry was used to analyze changes in mean fluorescent intensity (MFI) based on changes in concentration of CLIP on activated B-lymphocytes after treatment with the various formulations of vitamin D3. The human formulation of activated vitamin D (calcitriol) had the most dramatic reduction in CLIP density at an MFI of 257.3 [baseline of 701.1 (*P* value = 0.01)]. Cholecalciferol and alfacalcidiol had no significant reduction in MFI at 667.7 and 743.0, respectively. Calcitriol seemed to best reduce CLIP overexpression in this ex vivo model. Bioactive vitamin D3 may be an effective compliment to other B cell suppression therapeutics to augment downregulation of nonspecific inflammation associated with many autoimmune disorders. Further study is necessary to confirm these findings.

## 1. Introduction

B cell engagement by mitogens has been shown to result in the activation of B cell dependent inflammatory pathways. This interaction helps to “jump start” the innate immune response [1]. Emerging evidence suggests that vitamin D may also play a pivotal role in the successful downregulation of this aspect of the immune response as the host defense system transitions from innate to adaptive immunity via inactivation of nonspecifically activated, CLIP^+^ B cells [2–4]. In addition, bioactive vitamin D3 has been shown to inhibit mitogen-induced IgM production by B-lymphocytes as the affected host shifts from a nonspecific to specific immune response. This has been demonstrated to occur in a time-dependent fashion [2].

Furthermore, it has been previously established that mitogens bind to toll-like receptors on B cells, leading to nonspecific B cell activation associated with increased expression of class II-associated invariant chain peptide (CLIP) and polyclonal expansion of B cells into immunoglobulin (IgM) secreting (plasma) cells [1]. Class II-associated invariant chain peptide (CLIP), a polypeptide involved in the formation and transport of major histocompatibility complex (MHC) class II protein, also known as CD74, binds to the MHC class II groove and shields the epitope binding site until the MHC receptor is fully assembled [1, 5]. The purpose of CLIP is to prevent self-peptide fragments from binding to the receptor prior to MHC II localization within the endosome/lysosome after activation by foreign antigen (Ag) [1, 6]. Under normal conditions, in this special endosome called the major histocompatibility complex (MHC) II compartment, cathepsin S cleaves the invariant chain, leaving a shorter CLIP bound to the MHC II complex [7].

In presence of antigen peptide fragments, human leukocyte antigen- (HLA-) DM, an intracellular protein involved in foreign peptide presentation by MHC class II, interacts with the MHC II complex, leading to the release of CLIP and allowing the antigenic peptides to be bound via one of its epitopes [1, 8]. This is a key step in activation of the innate immune system. MHC II complexes with bound antigen and is subsequently transported to the cell membrane for presentation by antigen presenting cells (APCs), such as macrophages, dendritic cells, and B-lymphocytes [1, 8]. The antigen peptide-MHC class II complexes are then transported to the plasma membrane of the APCs, where they are recognized by T and B-lymphocytes as the host defense system activates its adaptive immune system [1, 5].

In some patients, there appears to be a failure to downregulate this initial inflammatory process [1, 9, 10]. This failure to turn off the acute immune response may lead to postinflammatory, persistent systemic inflammatory disorders, such as rheumatoid arthritis and hay fever, or a local inflammatory condition like psoriasis [1, 11].

As activated vitamin D3 has been demonstrated to be a powerful modulator of the immune system, it may play a significant role in the downregulation of autoimmune inflammatory disorders via suppression of nonspecifically activated immune cells [3, 4, 12–14]. In other words, vitamin D may be potentially useful in the amelioration of the proinflammatory pathway. In fact, vitamin D has previously been shown to reduce polyclonal B cell expansion. Chen et al. evaluated the effects of activated vitamin D3 on B cells and demonstrated its inhibition of ongoing proliferation of activated B cells [3]. This suggests that it may play a role in the attenuation of some chronic inflammatory conditions, which provides a rationale for its use as a component of B cell depletion therapy [3, 15, 16].

Consequently, the correction of vitamin D deficiency to optimal or therapeutic levels may play a significant role in the reduction of postinfectious, chronic inflammatory conditions and other B cell-mediated autoimmune disorders [17]. However, the best formulation or preparation of vitamin D3 which provides the most therapeutically appropriate benefit remains a subject of debate. In this study, we evaluated the effect of vitamin D3 on activated mice splenic B cell using various formulations and concentrations on the intensity of CLIP expression after CpG-mitogen stimulation. We hypothesize that bioactive vitamin D downregulates the density of CLIP expression as well as reducing polyclonally activated splenic B cells, thereby reducing the proinflammatory response.

## 2. Materials and Methods

Thirty C57Black/6 mice were obtained from the Jackson Laboratories (Bar Harbor, ME, USA) and divided into 5 groups of 6. All animal experiments were conducted according to the guidelines for animal use approved by the Texas A&M University Health Sciences. The average weight at the time of the experiments for the mice was 25 grams. All procedures involving the mice were performed in accordance with the guidelines of the Institutional Animal Care and Use Committee (IACUC) of the Texas A&M University Health Sciences.

15G4 monoclonal antibodies (mAb) were used in these experiments, a mAb directed against mouse MHC-CLIP (I-A^b^ complex), only when CLIP is in the groove of mouse MHC class II I-A^b^ molecules (Santa Cruz Biotechnology, Santa Cruz, CA, USA). Phycoerythrin- (PE-) conjugated monoclonal anti-mouse B220 was also used and obtained from BD PharMingen (San Diego, CA, USA). Mouse anti-human CLIP (clone CerCLIP) was obtained from BD Biosciences (San Jose, CA, USA). A TLR-9 binding ligand, also known as a toll-ligand, CpG-oligodinucleotide (CpG-ODN) (Invivogen, San Diego, CA), was used to prime the mice.

Thirty C57BL/6 mice were injected with CpG-ODN using ~5 *μ*g/mouse, weighing ~25 g, equivalent to 5 *μ*M of peptide, and then separated into 5 groups containing 6 mice each. Forty-eight hrs after initial TLR stimulation with CpG-ODN, the mice were sacrificed and their spleens were harvested and passed through a nylon mesh to recover single cell suspensions. Each group of mixed, resting B cells and CpG-ODN activated splenocytes were further subdivided into three groups and then treated with different vitamin D formulations of vitamin D3 (human calcitriol, exogenously synthesized calcitriol, cholecalciferol, and alfacalcidiol) at the following concentrations: 0.1 mg/dL, 1 mg/dL, and 10 mg/dL. DMSO was used as a control. The isolated cell populations were then allowed to incubate for 15 hrs before mAb staining with anti-mouse MHC-CLIP and anti-mouse B220. The cell preparation was analyzed using flow cytometry to determine the density of CLIP expression on the surface of the B cell populations by measuring the mean fluorescent intensity [MFI] (Beckman Coulter Excel or Coulter FC500 flow cytometer Beckman Coulter, Fullerton, CA, USA) of each treatment group.

One-sample *t*-test, 2-sample *t*-test, and ANOVA were used to analyze the data using MiniTab 17.0 software, State College, PA. One-sample *t*-test and 2-sample *t*-test were performed using MiniTab 17.0 software. ANOVA was used to create a generalized linear model using SAS 9.3, Cary, NC. A *P* value of < 0.05 was considered to be statistically significant.

## 3. Results

**Figure 1:**
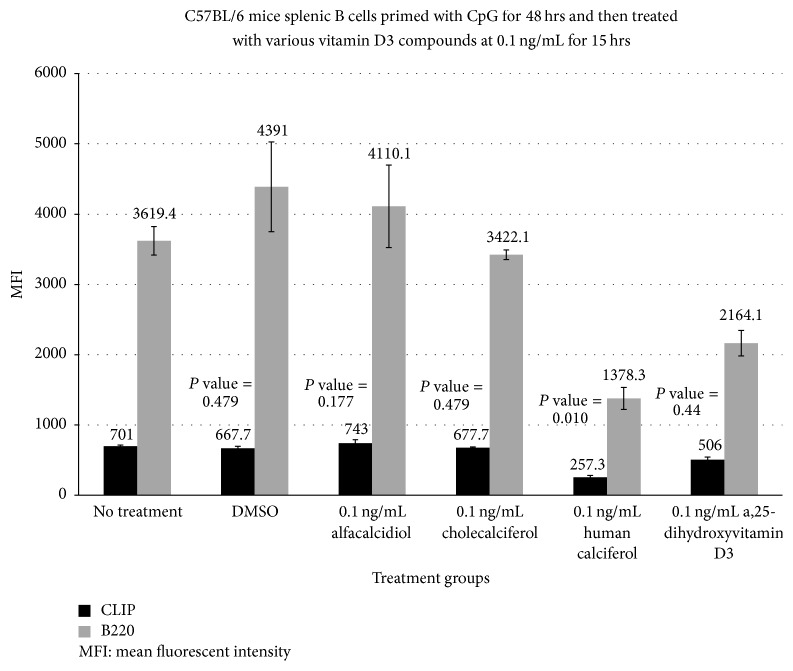
This bar graph shows the effects of various vitamin D3 compounds at a low concentration of 0.1 ng/mL on the mean fluorescent intensity (MFI) of CLIP^+^ B cells after TLR-engagement. This figure demonstrates the changes in MFI of CLIP and CLIP^+^ B220 subsets of B cells after CpG-induced TLR-9 activation. These results suggest that the active formulation of vitamin D3 may lead to a statistically significant reduction in CLIP expression on polyclonally activated splenic B cells. Administration of the human calcitriol of vitamin D3 (*P* = 0.01) resulted in the greatest overall reduction in the MFI (257 versus a baseline of 701.1) for CLIP expression on stimulated B cells.

**Figure 2:**
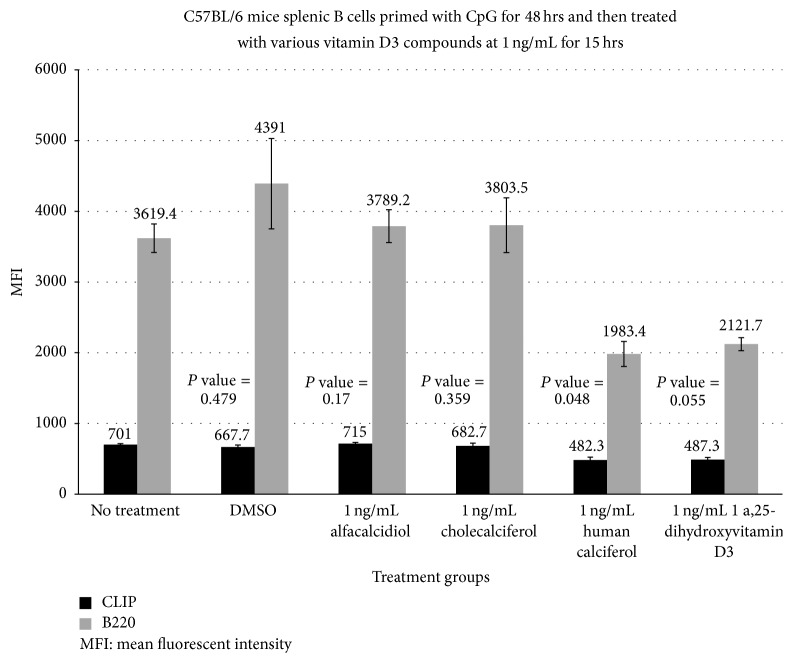
Bar graph showing the effects of 1 ng/mL of various vitamin D3 compounds on the distribution of TLR-activated CLIP^+^ and B220 subsets of B cells. This figure demonstrates the changes in MFI of CLIP and CLIP^+^ B220 subsets of B cells 15 hrs after administration of different compounds of vitamin D3 versus no treatment or DMSO (control). These results suggest that the active formulation of vitamin D3 may lead to a statistically significant reduction in CLIP expression on polyclonally activated splenic B cells. Administration of the human calcitriol of vitamin D3 (*P* = 0.048) resulted in the greatest overall reduction in the MFI (482.3 versus a baseline of 701.1) for CLIP expression on stimulated B cells. Administration of the active form of vitamin D3 again resulted in statistically significant reduction in CLIP expression on CpG-activated B cells.

**Figure 3:**
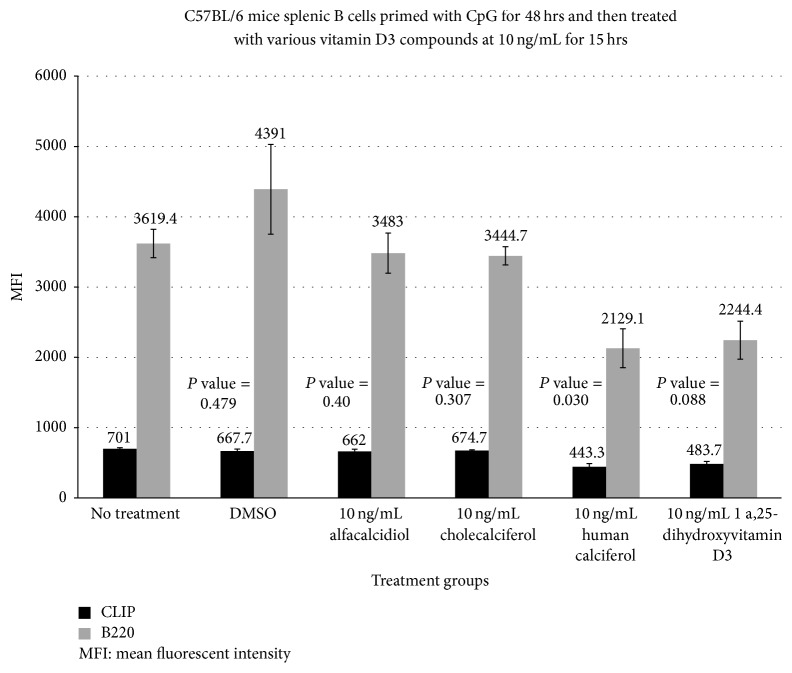
This bar graph also shows the effects of different vitamin D3 compounds at a concentration of 10 ng/mL on the MFI of CLIP expression and CLIP^+^ B220 subsets of B cells after TLR-activation. This graph compares the changes in MFI of CLIP and CLIP^+^ B220 subsets of B cells 15 hrs after administration of different compounds of vitamin D3 versus no treatment or DMSO (control). These results suggest that exogenously produced bioactive 1,25(OH)^2^D3 may be needed in higher doses of activated vitamin D3 in order to reduce the level of CLIP expression on polyclonally activated B cells (*P* = 0.055). Administration of human calcitriol again resulted in most significant reduction in CLIP expression on polyclonally activated B-lymphocytes.

The human activated vitamin D3 (calcitriol) formulation at 0.1 ng/mL (Figure 1) had the most dramatic reduction in the MFI of CLIP on activated B cells at 257.3 compared with a baseline MFI of 701.1 (*P* value = 0.01). Cholecalciferol and alfacalcidiol had no significant reduction in MFI at 667.7 and 743.0, respectively, fifteen hrs after administration. The effect appeared to be dose dependent with a less dramatic effect at higher doses (MFI of 257.3 versus 482.3 and 443.3) at 0.1, 1.0 (Figure 2), and 10 mg/dL (Figure 3), respectively. TLR-9 activation with CpG-ODN caused enhanced ectopic CLIP expression on activated B-lymphocytes at baseline.

In this study, CpG-ODN stimulation of mouse splenocytes resulted in a time-dependent increase in exogenous CLIP expression along with MHC class II complexes on B cells, as determined by immunofluorescent staining with an anti-mouse CLIP/class II-specific antibody versus anti-mouse B220 (Figure 1, bar graph). Upon analysis of the variations in percent of CLIP^+^ B cells over time with the changes in geometric mean fluorescent intensity (MFI), we observed that the relative number of CLIP molecules per cell decreased in response to administration of activated vitamin D3 [human and exogenous 1,25(OH)^2^D3] and incubation over the 15-hour time period, as well as the percentage of CLIP^+^ B cells over total number of B cells decreased in response to activated vitamin D3.

## 4. Discussion

**Figure 4:**
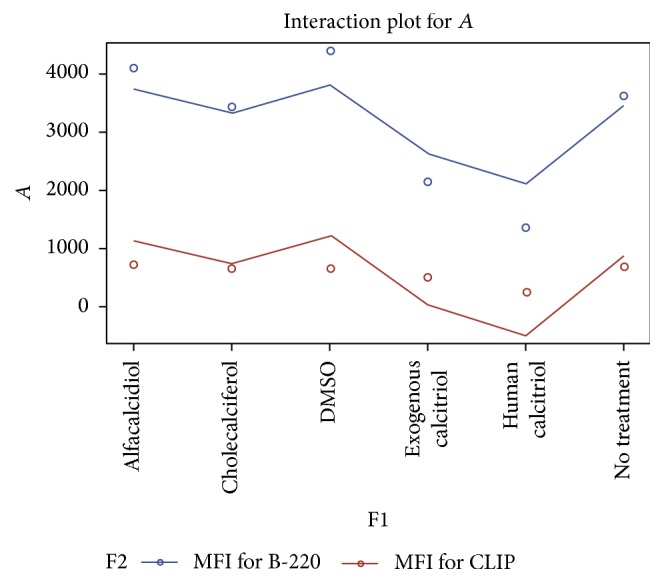
This figure compares the impact of the various vitamin D compounds at 0.1 mg/dL on the density of cell surface CLIP expression and B220 subsets of B cells 15 hrs after treatment using the vitamin D3 compounds versus nontreatment or DMSO. These results suggest that the active formulation of vitamin D3 once again leads to a statistically significant reduction in CLIP expression on polyclonally activated splenic B cells even at a higher concentration of 10 mg/dL. Administration of the human calcitriol of vitamin D3 (*P* = 0.0181) resulted in the greatest overall reduction in the MFI (257.3 versus a baseline of 701.1 and 667.7 for the control) of CLIP expression on mitogen stimulated B cells.

**Figure 5 fig5:**
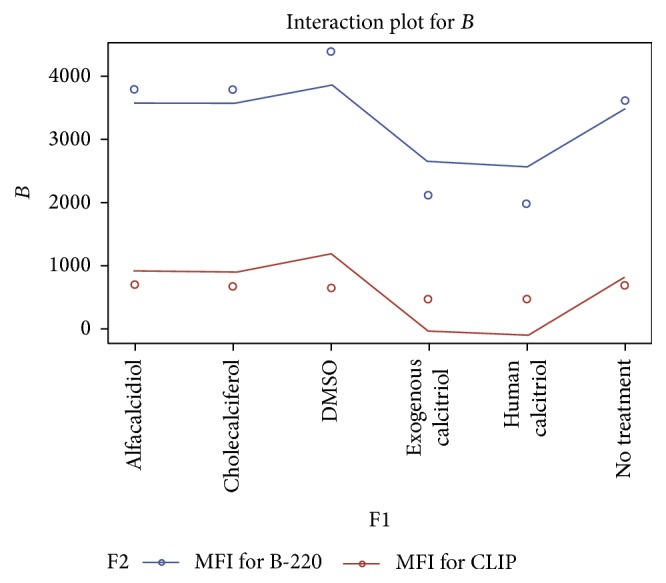
This figure compares the impact of the various vitamin D compounds at 1.0 mg/dL on the density of cell surface CLIP expression and B220 subsets of B cells 15 hrs after treatment using the vitamin D3 compounds versus nontreatment or DMSO. These results suggest that the active formulation of vitamin D3 once again leads to a statistically significant reduction in CLIP expression on polyclonally activated splenic B cells even at a higher concentration of 10 mg/dL. Administration of the human calcitriol of vitamin D3 (*P* = 0.0181) resulted in the greatest overall reduction in the MFI (482.3 versus a baseline of 701.1 and 667.7 for the control) of CLIP expression on mitogen stimulated B cells.

**Figure 6:**
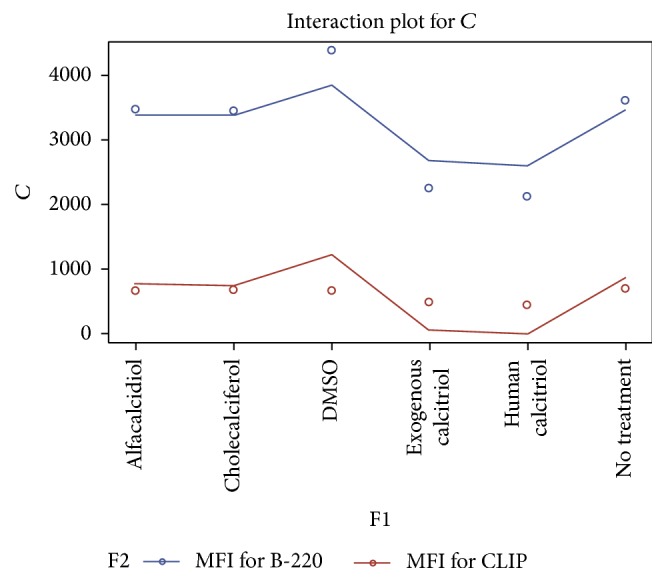
This linear graph demonstrates the impact of the various vitamin D compounds at 10.0 mg/dL on the density of cell surface CLIP expression and B220 subsets of B cells 15 hrs after treatment using the vitamin D3 compounds versus nontreatment or DMSO. These results suggest that the active formulation of vitamin D3 once again leads to a statistically significant reduction in CLIP expression on polyclonally activated splenic B cells even at a higher concentration of 10 mg/dL. Administration of the human calcitriol of vitamin D3 (*P* = 0.0181) resulted in the greatest overall reduction in the MFI (443.3) versus a baseline of 701.1 for no treatment and 667.7 for the control of CLIP expression on mitogen stimulated B cells.

In these experiments, C57BL/6 mice were injected with CpG-ODN and incubated for 48 hrs to stimulate polyclonal B cell expansion. The cells were then treated with bioactive and precursor formulation of vitamin D3 at varying concentrations to determine if treatment of this nature had any effect on the density of CLIP expression. CpG stimulation induced an approximately 8-fold increase in the number of CLIP^+^ B cells from baseline, resting B cells. A significant decrease in ectopic CLIP expression on the surface of activated B cells was observed fifteen hours after treatment with activated vitamin D3 (Figures 4 [Fig fig5]–6) [1, 16].

To rule out the possibility that ectopic CLIP resulted solely from coincident, increased levels of nascent MHC class II on the activated B cells, we counterstained activated B cells with an MHC class II anti-human HLA-DR antibody [1]. The increase in cell surface CLIP levels in response to CpG-ODN did not correspond with the TLR-dependent changes in MHC class II, suggesting that TLR-mediated ectopic CLIP expression is not merely the consequences of randomly increasing levels of cell surface MHC class II. Actually, cell surface CLIP is considered to be an indicator of immaturity in antigen presenting cells until they are activated by peptide antigen specific MHC receptor engagement, at which point cell surface CLIP expression decreases [1, 18–20].

As B cell-specific antigen receptor (BCR) engagement results in signals that increase the acidity in the lysosomes, the BCR subsequently works in conjunction with 1, 25-dihydroxyvitamin D3 to suppress the expression of CLIP on activated B cells [3, 5, 6]. We directly evaluated the effects of TLR stimulation on CLIP expression versus B2220 presence on the cell surface. We used anti-Ig stimulation as a known activator for B220 receptor signaling and compared levels of ectopic CLIP and percentage of CLIP^+^ B cells after TLR-9 dependent B cell activation versus stimulation through the B cell antigen receptor [1]. As predicted, we observed significantly less ectopic CLIP per cell by measuring geometric mean fluorescent intensity (MFI) in cell populations in which there was no TLR stimulation (resting B cells).

Similarly, the percentage of CLIP^+^ B cells after vitamin D3 treatment was reduced significantly relative to the percentage of CLIP^+^ B cells after initial TLR-9 activation (Figures 4 [Fig fig5]–6). DMSO (control), cholecalciferol, and alfacalcidiol treatment resulted in no relevant decrease in CLIP expression as measured by changes in relative MFI. These results indicate that activated vitamin D3 versus its precursor cholecalciferol and alfacalcidiol formulations resulted in a statistically significant decreased level of expression of surface CLIP on activated B cells. Therefore, unabated TLR stimulation by CpG-ODN or other mitogens, which is not followed by active vitamin D receptor engagement, may significantly increase the percentage of cell surface, CLIP-positive B cells, and the relative amount of CLIP expressed per cell leading to ongoing nonspecific inflammation [1, 17, 21, 22].

Our data support the fact that nonspecifically activated B cells, which bear increased levels of ectopic CLIP, are important for promoting nonspecific, proinflammatory immune activation. Treatment with an anti-inflammatory agent, such as the activated form of vitamin D3, might decrease the density of CLIP on activated B cells [22, 23]. This may cause or help facilitate the transition of the host defense system from nonspecific to specific adaptive immunity.

As mentioned earlier, accumulating research data suggest that 1,25(OH)^2^D3-mediated signaling is important in the regulation of protective inflammatory responses against harmful pathogens [23, 24]. In addition to inhibiting expression of nonspecific immunoglobulin (IgM) by B cells, activated vitamin D3 has been shown to enhance production of anti-inflammatory mediators as well as suppressing the expression of IL-2 receptors on B cell blasts [3]. In order to optimally regulate immune function, research suggests that serum levels of cholecalciferol must be greater than 30 ng/mL in order to undergo local, paracrine conversion to calcitriol [24–28]. The biofeedback of locally produced activated vitamin D3 creates a condition whereby antigen-responsive B cells are able to simultaneously engage in the induction of adaptive immune responses to specific antigenic stimuli. Therefore, activated vitamin D3 at appropriate levels may serve as an important contributor to anti-inflammatory processes, as it may help to dampen both acute and chronic, nonspecific inflammatory response pathways and prevent or enhance amelioration of some autoimmune conditions [29–32].

In this study, we observed that the relative number of CLIP molecules per cell decreased in response to administration and incubation with activated vitamin D3 [human calcitriol and exogenous 1, 25 (OH)^2^ vitamin D3] over the treatment period. Furthermore, the MFI for the total number of CLIP^+^ B220 cells was noted to decrease in response to activated vitamin D3. This suggests that calcitriol may directly, or indirectly, play a vital role in augmenting humoral immunity as well as advances in B cell depletion therapies for certain autoimmune conditions. Based on our preliminary study, plus several others which suggest that dampening chronic immune activation responses using innovative therapeutics may be beneficial, vitamin D3 may be a useful adjunct to use with other B cell depletion therapies for the various autoimmune diseases and disorders characterized by chronic inflammation. However, the mechanism by which this B cell depletion takes place is still being elucidated and is an area of ongoing research [33–40]. Furthermore, additional study will be necessary to determine the most salient aspects of this potential intervention before specifically testing this type of therapy to treat any specific autoimmune-related medical condition.

## 5. Conclusion

B cell depletion therapy has been suspected to be a useful modality in dampening of chronic inflammatory conditions. As activated vitamin D was demonstrated to suppress CLIP expression on the surface of polyclonally activated splenic B cells after nonspecific priming with mitogen, it may be a useful adjunct to further explore and study to enhance the success of B cell depletion. Our study suggest that bioactive vitamin D3 may be helpful in the amelioration of some aspect of inflammation, as increased CLIP expression on polyclonally activated B-lymphocytes has been linked to acute nonspecific inflammatory processes. Plasma levels of vitamin D3 in the range of ≥30 to 40 ng/mL may help augment this immune downregulation process and/or facilitate the reversal of some acute and chronic autoimmune disorders through suppression of some aspects of the proinflammatory pathway [41–44]. As activated vitamin D3 has been demonstrated to be a powerful modulator of the entire immune system, it may play a potentially significant role in the downregulation of nonspecifically activated, Ig-M secreting B cell activation through suppression of CLIP expression on polyclonal B-lymphocytes. This may be used to compliment other mechanisms and strategies through which B-lymphocyte depletion therapies may help to diminish chronic immune activation. Nevertheless, further study is necessary to confirm the results of our preliminary findings.
